# Hallmarks of Cancer-Related Newly Prognostic Factors of Oral Squamous Cell Carcinoma

**DOI:** 10.3390/ijms19082413

**Published:** 2018-08-16

**Authors:** Tomonori Sasahira, Tadaaki Kirita

**Affiliations:** 1 Department of Molecular Pathology, Nara Medical University, 840 Shijo-cho, Kashihara, Nara 634-8521, Japan; 2 Department of Oral and Maxillofacial Surgery, Nara Medical University, 840 Shijo-cho, Kashihara, Nara 634-8521, Japan; tkirita@naramed-u.ac.jp

**Keywords:** oral cancer, hallmarks of cancer, prognostic factors

## Abstract

Head and neck cancer, including oral squamous cell carcinoma (OSCC), is the sixth leading malignancy worldwide. OSCC is an aggressive tumor and its prognosis has exhibited little improvement in the last three decades. Comprehensive elucidation of OSCC’s molecular mechanism is imperative for early detection and treatment, improving patient survival. Based on broadly accepted notions, OSCC arises from multiple genetic alterations caused by chronic exposure to carcinogens. In 2011, research revealed 10 key alterations fundamental to cancer cell development: sustaining proliferative signaling, evading growth suppressors, avoiding immune destruction, activating invasion and metastasis, tumor-promoting inflammation, enabling replicative immortality, inducing angiogenesis, genome instability and mutation, resisting cell death, and deregulating energetics. This review describes molecular pathological findings on conventional and novel hallmarks of OSCC prognostic factors. In addition, the review summarizes the functions and roles of several molecules as novel OSCC prognosticators.

## 1. Introduction

Head and neck cancer, including oral squamous cell carcinoma (OSCC), is the sixth leading cancer worldwide, with an estimated 300,400 cases and 145,400 OSCC-related deaths occurring in 2012 [1]. OSCC is one of the leading causes of morbidity and mortality in Melanesia, South Central Asia, and Central and Eastern Europe [1]. It arises anywhere in the oral cavity, including the tongue, upper and lower gingiva, oral floor, palate, and buccal mucosa. Despite advances in cancer diagnosis and treatment, the overall 5-year survival rate for OSCC remains the lowest among malignancies and, in fact, has been <50% for the last three decades [2]. Anatomically, OSCC of the tongue and gingiva is prone to invade the deeper muscles and jawbone, respectively. In addition, OSCC tends to cause cervical lymph node metastasis because the lymphatic vessels in the oral cavity are rich and comprise numerous anastomoses [3]. Furthermore, OSCC often causes dysfunctions in chewing and swallowing, as well as speech and esthetic disorders, which can worsen patients’ quality of life [4]. Hence, the recurrent potential of OSCC is considered to be closely associated with the local expansion and nodal metastasis of tumor cells. However, specific molecular OSCC prognosticators have only been partially identified.

**Figure 1:**
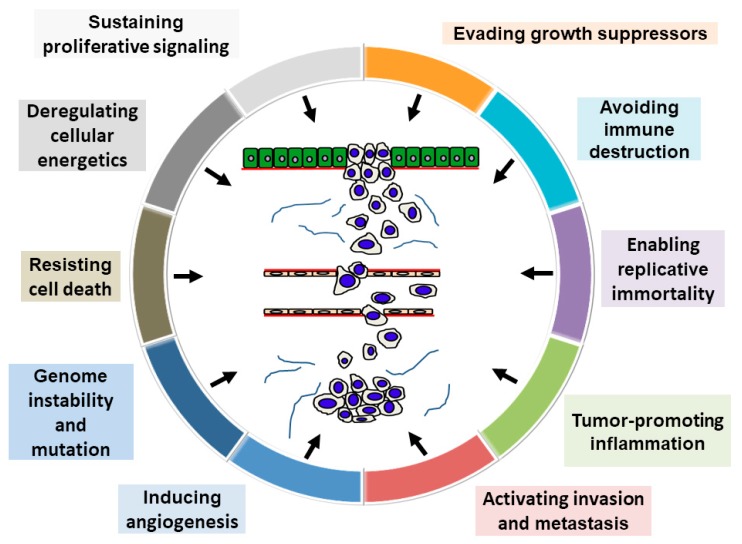
Schema of the hallmarks of cancer. This schema presents the 10 hallmark capabilities as follows: sustained proliferative signals, evasion of growth suppressors, resistance to cell death, replicative immortality, induction of angiogenesis, activation of invasion and metastasis, avoidance of immune destruction, deregulation of cellular energetics, genome instability and mutation, and tumor-promoting inflammation. Adapted reproduction with permission from Ref. [4].

Recently, Hanahan and Weinberg [5] proposed that the following “10 hallmarks of cancer” are pivotal in tumor progression: sustaining proliferative signaling, evading growth suppressors, avoiding immune destruction, activating invasion and metastasis, tumor-promoting inflammation, enabling replicative immortality, inducing angiogenesis, genome instability and mutation, resisting cell death, and deregulating energetics. Figure 1 presents a schematic illustration of this model, assuming it applies to OSCC prognostication. This review aims to summarize the general major OSCC prognosticators based on the hallmarks of cancer. In addition, the review describes our latest molecular pathological findings regarding OSCC prognostic predictors.

## 2. Hallmarks of Cancer-Related Conventional Principal Prognostic Factors of OSCC

### 2.1. Sustaining Proliferative Signaling

Healthy cells regulate growth signals through soluble and membrane-bound growth factors; however, cancer cells are characterized by autonomous, chaotic growth because of deregulated growth signals [6]. The epidermal growth factor (EGF) family are transmembrane tyrosine kinase receptors comprising epidermal growth factor receptor (EGFR) or human epidermal growth factor receptor 1 (HER1), HER2, HER3, and HER4 [7]. Studies have established a marked correlation between the EGFR, phosphorylated EGFR (pEGFR), HER2, or HER4 expression and the poor survival of OSCC patients [8,9,10]. The overexpression of cyclin D1, a cell-cycle regulator from G_1_ to S-phase, also indicates low survival [11]. C-Met is another transmembrane tyrosine kinase receptor that is associated with the poor prognosis in OSCC patients through the activation of matrix metalloproteinase matrixins (MMP)-1, -2, and -9 [12]. In addition, the signal transducer and activator of transcription (STAT) family members are cytoplasmic transcription factors, and a recent study suggested that cases with phosphorylated STAT3 (pSTAT3) expression indicated the worst OSCC prognosis [13]. Furthermore, STAT3 and c-Met co-expression is involved in OSCC progression [14].

### 2.2. Evading Growth Suppressors

In cancer cells, several tumor-suppressor genes associated with antigrowth signals are inactivated by mutation, deletion, and methylation. It is well established that p53 is a genome guardian and plays a pivotal role in regulating the cell cycle, cellular differentiation, DNA repair, and apoptosis [15]. Somatic mutations in p53 are detected in 60–80% of OSCC and in early 10% of oral dysplasia [6]. Recently, Genome Wide Association Study data have shown that p53 is usually mutated in cases with human papillomavirus-negative OSCC [16]. Recently, the p53 mutation grading system, which classifies low-risk missense mutations, high-risk missense mutations, and other mutations have developed in head and neck cancer containing OSCC, and subgroups of high-risk p53 mutations are associated with decreased sensitivity to cisplatin, distant metastasis, extranodal extension, and poor prognosis [17,18,19]. The overall survival of p53-mutant OSCC patients is also markedly worse than that of patients with p53 wild-type [20]. In addition, the reduction of cell-cycle regulator p16 or p21 expression levels markedly correlated with poor prognosis [21,22]. Phosphatase and tensin homolog (PTEN) acts as a tumor repressor through negative feedback of the phosphoinositide 3-kinase (PI3K)–Akt–mammalian target of rapamycin (mTOR) pathway [23]. Moreover, PTEN inhibits insulin signaling by indirectly suppressing the phosphorylation of mitogen-activated protein kinase (MAPK) and blocking insulin receptor substrate 1 (IRS-1) phosphorylation [23]. In OSCC, the absence of PTEN expression predicts unfavorable prognosis [24]. Incidentally, PTEN is inactivated due to gene methylation because *PTEN* mRNA restoration is observed post-treatment with 5-aza-2′-deoxycytidine (5-Aza-dc), a demethylation reagent, in human OSCC-derived cells [25].

### 2.3. Avoiding Immune Destruction

Among lymphocytes, CD8^+^ cytotoxic T cells (CTL) serve as antitumor immunity cells in cooperation with CD4^+^ T helper type 1 cells (Th1 cells). However, chemokines can recruit CD4^+^ Th2 cells and CD4^+^ T regulatory (Treg) cells into the tumor microenvironment, causing inhibited CTL antitumor responses [26]. Previously, we reported that the melanoma inhibitory activity 2 (MIA2) in OSCC is promoted by a disturbance in the tumor immunity through the suppression of CTL, Th1 cells, and CD40L^+^ and granzyme B^+^ T lymphocytes and relative increment in Treg cells [4,27]. In addition, tumor-infiltrating myeloid cells, with the co-expressing macrophage marker CD11b and the neutrophil marker Gr1, have been reported to suppress CTL and natural killer (NK) cell activity [5]. Reportedly, chemokine (C-X-C motif) ligand 9 (CXCL9) is an interferon-γinducible chemokine, and higher CXCL9 serum levels are independent predictors of the overall and disease-free survival in OSCC patients OSCC [28]. Furthermore, secretion and expression levels of interleukin (IL)-8 are implicated in poor clinical outcomes through the generation of CD163-positive M2 macrophages in OSCC [29]. Programmed cell death ligand 1 (PD-L1) and its receptor PD-1 play a central role in tumor immune escape and the formation of a tumor microenvironment [30]. Overexpression of PD-L1 on tumor cells and PD-1 on tumor-infiltrating lymphocytes is correlated with poor disease outcome in various human cancers [31]. Antagonists of PD-1 and PD-L1 have demonstrated clinical utility in several types of advanced malignancies [32]. In OSCC, PD-L1/PD-1 expression is a useful predictor for nodal metastasis and poor prognosis in OSCC cases [33]. 

### 2.4. Activating Invasion and Metastasis

OSCC metastasis primarily occurs through the cervical lymph nodes on the affected side. The following sequential steps are fundamental to invasion and metastasis of cancer cells:

1. Declined adhesion and detachment of cancer cells;
2. Disruption of the basal membrane;
3. Acquisition of cancer cell movement and stromal infiltration;
4. Intravasation;
5. Intravascular migration;
6. Extravasation;
7. Cancer cell growth in the metastatic foci [6].

In addition, cancer cells are unbound by adhesion molecule abnormalities. E-cadherin, which plays a pivotal role in maintaining cell-to-cell adhesions in normal epithelial cells, correlates with poorer OSCC prognosis [34]. Likewise, integrins, which are heterodimeric cellular transmembrane proteins, are also crucial adhesion molecules mediating cell-to-cell and cell-to-extracellular matrix interactions [6]. The overexpression of integrin αvβ6 is an unfavorable clinical prognostic factor in OSCC patients [35]. In addition, the prognosis of patients with integrin-α7 expression is markedly worse than that of patients without the integrin α7 expression [36]. MMPs are secretory proteolytic enzymes which are involved in extracellular matrix modulation and the destruction of the basement membrane [6]. Recent studies suggested that OSCC cases with expression of MMP-7, -11, -13, or -21 exhibit a markedly lower survival rate [37,38,39,40]. Similarly, the epithelial–mesenchymal transition (EMT), characterized by a reduction of epithelial propensities and the acquisition of a mesenchymal phenotype, plays a pivotal role in the invasion and metastasis of cancer cells. Reportedly, critical EMT transducers are transforming growth factor-β (TGF-β), Wnt, Notch, interleukin-like EMT-inducer, hepatocyte growth factor, EGF, and platelet-derived growth factor (PDGF) [41]. Moreover, tumor cells with induced EMT exhibit a decline in the epithelial cell-to-cell attachment by repression of E-cadherin, ZO-1, occludin, and others, and the overexpression of mesenchymal markers, including smooth muscle actin, vimentin, N-cadherin, and desmin [41]. Furthermore, the upregulation of transcription factors, such as Snail, Slug, Twist, and ZEB1/2, is fundamental to retention of the EMT status in cancer cells [6,41]. Consequently, EMT gain contributes to poorer outcomes for OSCC patients [42].

### 2.5. Tumor-Promoting Inflammation

Apparently, inflammatory cells promote the development, advancement, and metastasis of cancer by producing tumor-promoting cytokines. Inflammation can alter the tumor microenvironment by inducing growth, survival, proangiogenic factors, and reactive oxygen species. It can also modify the extracellular matrix, thereby promoting angiogenesis, invasion, and metastasis [5]. In addition, stromal fibroblasts may play a vital role in the desmoplastic reaction to cancer by secreting the extracellular matrix. Eltohami et al. [43] reported that the systemic inflammatory score (SIS), which is based on the sum of albumin and the lymphocyte-to-monocyte ratio, is closely associated with local progression (T grade), clinical stage, tumor depth, perineural invasion, extranodal extension, and poor survival. Cyclooxygenase-2 (COX-2) is a pro-inflammatory enzyme that converts arachidonic acid to prostaglandins, promoting invasion and OSCC cell metastasis [44]. The COX-2 expression is an independent prognostic factor by immunohistochemistry [45]. Furthermore, the IL-6 overexpression, which is a pleiotropic cytokine, in OSCC cells is a good predictor of poor response to chemo/radiotherapy and poor prognosis [46]. 

### 2.6. Enabling Replicative Immortality

Telomeres are tandemly repeated DNA sequences with 5′-TTAGGG-3′ present at the linear ends of chromosomes; these elements are bound to specific proteins, including telomeric repeat factors 1 and 2 (TRF1 and TRF2), TRF1-interacting nuclear factor 2 (TIN2), repressor activator protein 1 (RAP1), protection of telomeres (POT1), and POT1-interacting protein (TPP1) [47]. Reportedly, the telomere length is maintained by telomerase, and its activity is regulated by the expression of human telomerase reverse transcriptase (hTERT), as the catalytic subunit of telomerase [48]. In addition, telomere plays a crucial role in OSCC tumorigenesis and progression. Telomere dysfunction is a valid predictor of radioresistance in OSCC cells [49]. Moreover, high-expression levels of hTERT are involved in the oral carcinogenesis at an early phase and unfavorable outcomes for OSCC patients [50]. Furthermore, TRF2 immunopositivity, which interacts with the distal ends of chromosomes to protect telomere, is a good marker of poor prognosis [51]. 

### 2.7. Inducing Angiogenesis

Although cancer causes tissue hypoxia, the provision of oxygen and nutrients and withdrawal of waste products are essential for tumor cells. Angiogenesis, the formation of new blood vessels, and lymphangiogenesis, the proliferation of new lymphatic vessels, are essential for the growth, invasion, and metastasis of cancer cells [52]. A recent study suggested that antiangiogenic gene therapy might be useful for the prevention and early treatment of malignancies [53]. Typically, compared to normal vessels, neoplastic vessels are leaky and dilated, lack pericytes, and can be attenuated for anticancer drug delivery [52,54]. However, high microvessel density (MVD) and lymphovessel density (LVD) are markedly related to T grade, clinical stage, nodal metastasis, local recurrence, and poor outcomes [52]. In addition, family members of vascular endothelial growth factor (VEGF), including VEGF-A, -C, and -D, play a central role in the tumor angiogenesis and lymphangiogenesis [6,52]. In OSCC, expression levels of VEGF-A, -C, or -D are strongly related to only angiogenesis/lymphangiogenesis but also poorer outcomes [52,55,56]. 

### 2.8. Genome Instability and Mutation

Broadly, OSCC arises from multiple genetic and epigenetic alterations triggered by the chronic exposure to carcinogens such as alcohol, smoking, toxic chemical substance, viral infections, and inflammation; these genetic alterations include deletions, point mutations, promoter methylation, and oncogene amplification. They also inactivate tumor-suppressor genes [6]. Loss of heterozygosity (LOH) on chromosomes 3p, 9p (inactivation of *p16*), and 17p (inactivation of *p53*) correlates to early-phase oral carcinogenesis; conversely, genetic alterations on chromosomes 4q, 8p, 11q, and 13q correlates with late-phase oral carcinogenesis [4]. Recent genome-wide LOH and DNA copy-number aberration analysis revealed that regions on 4q, 8p, 9p and 11q play a vital role in the disease-specific survival of OSCC patients [57]. In addition, LOH on 1q21.3 is an independent prognostic factor in OSCC [58]. A high-throughput, genome-wide analysis using a next-generation sequencer provides a comprehensive platform to elucidate an overview of gene expression and mutation [59]. Recent reporting indicated that *p16*, *protocadherin FAT1*, *p53*, *caspase-8*, *PI3K*, *Notch1*, histone-lysine *N*-methyltransferase 2D (*KMT2D*), nuclear receptor binding SET domain protein 1 (*NSD1*), and *H-ras* are frequently mutated genes in head and neck SCC including OSCC [16]. Furthermore, the subgroup of OSCC cases with favorable clinical outcomes represents infrequent copy-number alterations combined with the activation of *H-ras* or *PI3K*, wild-type *p53*, and the inactivation of *caspase-8* and *Notch1* [16]. FAT1 regulates the migration and invasion of OSCC cells through the localization of β-catenin [60]. The PI3K/AKT/mTOR signaling pathway is associated with tumor growth, survival, metastasis, and treatment-resistant in OSCC [61]. Notch 1 has an oncogenic and tumor suppressive function in OSCC and its role is still controversial [62]. *KMT2D* and *NSD1* are chromatin remodeling gene and their alterations are strongly associated with sensitivity to aurora kinase inhibition and widespread genome hypomethylation in head and neck SCC, respectively [63,64]. High frequency of *H-Ras* mutation is detected in Asian populations associated with betel nut chewing [65]. 

### 2.9. Resisting Cell Death

The escape from apoptosis allows cancer cells to survive longer, providing more time for accumulating mutations [66]. Apoptosis can occur through both extrinsic (receptor-initiated death) and intrinsic (mitochondrial) pathways. The extrinsic pathway is triggered by the binding of tumor necrosis factor-γ (TNFγ) or Fas to TNF-related apoptosis-inducing ligand (TRAIL) or Fas ligand (FasL), thus activating caspase-8 and -3. The intrinsic pathway is regulated the by B-cell lymphoma-2 (Bcl-2) protein family, containing Bcl-2, Bcl-2-associated X protein (Bax), Bcl-2 homologous antagonist killer (Bak), and so on, through various stimuli, prompting the release of cytochrome C and enhancing caspase-9 and -3 [6,66]. The Bcl-2 overexpression is strongly related to the poor prognosis of OSCC patients [67]. Conversely, cases with the Bax or Bak overexpression exhibit a markedly better cancer-specific prognosis than those without the Bax or Bak expression [67,68]. Survivin is a member of the inhibitor of apoptosis protein (IAP) family and inhibits apoptosis by suppressing the activity of caspase-3, -7, and -9 [69]. Although survivin is expressed on cytoplasm in normal human oral keratinocytes, nuclear translocation of survivin by acetylation is observed in OSCC cells [70]. Nuclear staining of survivin is also associated with clinical stage and poor prognosis in OSCC patients [70]. Moreover, cellular IAP2 (cIAP2), other member of the IAP family, is involved in 5-fluorouracil (5-FU) resistance and shorter overall survival periods in OSCC [71].

### 2.10. Deregulating Energetics

Under aerobic conditions, healthy cells produce adenosine triphosphate (ATP) from glucose in the mitochondria and oxidative phosphorylation in the electron transfer system. In addition, ATP is produced through the anaerobic glycolytic pathway, which breaks down glucose in the cytoplasm and produces lactic acid under anaerobic conditions [6]. However, cancer cells can produce energy through aerobic glycolysis in the presence of oxygen (Warburg effect) [5,72]. Most cancer cells enhance glycolysis uncoupled with the oxidative phosphorylation, although glycolysis yields lower amounts of ATP from glucose than the oxidative phosphorylation [73,74]. Glucose transporter 1 (GLUT-1) is a transmembrane protein regulating the transport and metabolism of glucose, and elevated GLUT1 expression levels are observed in many malignancies [75,76]. In OSCC, the GLUT1 overexpression causes resistance to radiotherapy and chemotherapy and, thus, poor prognosis [77,78]. Another crucial cellular energetics-related factor is hypoxia-inducible factor 1α (HIF1α), which modulates target gene transactivation under hypoxic conditions [79]. Previously, we reported that [^18^F]fluoro-2-deoxyglucose-positron emission tomography (FDG-PET) imaging findings correlated with a therapeutic response to neoadjuvant chemoradiotherapy and immunohistochemical staining of HIF-1α in patients with advanced OSCC [80]. Furthermore, the HIF1-α expression correlated with OSCC angiogenesis, lymphangiogenesis, and poor prognosis [81,82]. 

## 3. Novel Prognosticators of OSCC

We investigated the hallmarks of cancer-related molecules to elucidate the molecular mechanism of cancer development, invasion, metastasis, and prognosis. Here, we describe the functions of 12 new prognostic factors in OSCC (Table 1).

### 3.1. miR-126

MicroRNAs (miRNAs) are noncoding small RNAs of approximately 18–25 nucleotides that regulate gene expression by binding to the 3′-untranslated region (UTR) of the target mRNA [6]. The biosynthetic process of the mature miRNA can be explained as follows. Primary miRNA (pri-miRNA) is processed in the nucleus into precursor miRNA (pre-miRNA) by the RNase Drosha and DiGeorge syndrome critical region gene 8 (*DCRG8*). Pre-miRNA is exported to the cytoplasm by exportin-5 and processed into mature miRNA by the RNase Dicer. After integration into the RNA-induced silencing complex (RISC), the mature miRNA regulates the target gene mRNA expression [6]. Recently, meta-analyses have revealed that the upregulation of 9 miRNAs (*miR-21*, *miR-455-5p*, *miR-155-5p*, *miR-372*, *miR-373*, *miR-29b*, *miR-1246*, *miR-196a*, and *miR-181*) and the downregulation of 7 miRNAs (*miR-204*, *miR-101*, *miR-32*, *miR-20a*, *miR-16*, *miR-17*, and *miR-125b*) are strongly correlated with poor prognosis in OSCC patients [83]. Long noncoding RNA (lncRNA), a class of non-protein coding transcripts longer than 200 nucleotides, is also associated with gene expression and cancer progression [84]. Among them, HOX transcript antisense RNA (*HOTAIR*) [84], metastasis-associated lung adenocarcinoma transcript 1 (*MALAT1*) [85] and *lncRNA H1* [86] are predictors of poor survival in OSCC. 

*miR-126* is an endothelial, cell-specific miRNA located in intron 7 of epidermal growth factor-like domain 7 (*EGFL7*), and its overexpression promotes vessel formation by repressing expression of sprouty-related protein-1 (Spred-1) in developmental angiogenesis [6,87]. Previously, we reported that miR-126 and its host gene, *EGFL7*, were downregulated by DNA hypermethylation in OSCC cells [88]. In addition, *miR-126* is a negative regulator of VEGF-A activation and promotes tumor cell growth in OSCC cells. In human OSCC specimens, a low *miR-126* expression was observed in 94 of 118 cases (79.7%) and was relevant to local tumor expansion (T grade), clinical stage, and nodal metastasis. Besides, the reduced *miR-126* expression correlated with tumor angiogenesis and lymphangiogenesis and poorer outcomes. Furthermore, multivariate analysis revealed that *miR-126* expression levels were independent prognostic factors for disease-free survival periods in OSCC. 

### 3.2. FOXC2

Forkhead box protein C2 (FOXC2) is a transcriptional regulatory factor which is essential to cardiovascular development, including vascular endothelial cell differentiation and lymphatic vessel formation [96]. Reportedly, FOXC2 is a tumor-progressive factor in various malignancies and is closely associated with metastasis and prognosis [97,98,99]. In addition, FOXC2 regulated EMT and the gain of multiple anticancer drug resistance in cancer cells [89,100]. In OSCC, immunostaining for FOXC2 was observed in 23.3% (38/163) of cases and markedly related to MVD [101]. In addition, cases with FOXC2-positive OSCC exhibited markedly poorer prognosis than those with FOXC2-negative OSCC. In the functional analysis under a coculture of human OSCC cells and vascular endothelial cells, FOXC2 promoted angiogenesis by enhancing VEGF-A expression. Furthermore, FOXC2 regulated the gene expression of prospero homeobox 1 (PROX1) in OSCC cells. Our results suggested that FOXC2 could be a novel angiogenic inducer in OSCC cells.

### 3.3. PROX1

PROX1 is a nuclear transcription factor associated with the embryonic development of multiple organs, including the central nervous system, heart, lymphatic system, skeletal muscles, lens, retina, and so on [102]. Reportedly, PROX1 plays various tumor-dependent functional roles, which reflect both the oncogenic potential and a tumor-suppressive role [90]. In addition, PROX1 promoted cell growth, angiogenesis, and sorafenib resistance in patients with HCC [102,91] and is associated with the lymphangiogenesis, metastasis, and poor prognosis in various malignancies [92,103,104]. However, high PROX1 expression is related to better prognosis for pancreatic and gastric cancer patients [105,106]. Thus, PROX1’s role in malignancies remains debatable. The PROX1 expression was found in 25.8% (42/163) of patients with OSCC by immunohistochemistry and was markedly associated with the local progression of the tumor (T classification), clinical stage, LVD, nodal metastasis, and expression levels of FOXC2 [101]. Besides, the survival and multivariate analysis revealed that PROX1 expression correlated with poor survival of OSCC patients. PROX1 also accelerated cell growth and lymphangiogeneis through VEGF-C activation in OSCC cells. Our findings indicated that PROX1 exhibits tumor-progressive function in OSCC. However, reportedly, the PROX1 reduction promoted OSCC cell proliferation [107]. Hence, further studies are warranted to elucidate the detailed molecular mechanisms underlying PROX1 in OSCC.

### 3.4. TANGO

Reportedly, *MIA* and *MIA2* are involved in OSCC tumor progression [27,52]. The expression of the *MIA* gene family members is reported in several malignancies [93]. Transport and Golgi organization protein 1 (*TANGO*) is one of the *MIA* gene family members and comprises a highly conserved Src homology 3 (SH3)-like domain [4]. *TANGO* could be a suppressor of the invasion and migration of malignant melanoma, colorectal cancer (CRC), and HCC [108,109]. However, the *TANGO* expression reportedly correlated with tumor progression, nodal and distant metastasis, and shortened disease-free survival in SCC of the esophagus, lung, and uterine cervix [93]. In OSCC, *TANGO* also regulated adhesion to OSCC cells, transendothelial migration, and tube formation of vascular and lymphatic vascular endothelial cells by activating the platelet-derived growth factor-β polypeptide (PDGFB) and neuropilin 2 [4,54]. Imatinib, an inhibitor of the PDGF receptor tyrosine kinase, might be useful for OSCC treatment because of decreased *TANGO* activity [4]. In addition, *TANGO* promoted migration and invasion while inhibiting apoptosis in human OSCC cells. We observed the *TANGO* expression in 35.1% (60/171) of OSCC specimens, markedly correlating with age, tumor progression (T grade) clinical stage, nodal metastasis, MVD, and LVD. Moreover, survival analysis elucidated markedly shorter disease-free survival periods in patients with the *TANGO* expression than in those without the *TANGO* expression. As the *MIA* gene family members are also secretory proteins [110], *TANGO* might be useful as a tumor marker detectable in the serum, saliva, urine, ascites and pleural fluid, and other samples [4,93]. Our findings suggested that *TANGO* exhibits tumor-progressive function in its activation of angiogenesis and lymphangiogenesis in OSCC.

### 3.5. HuD

Hu antigen D (HuD) serves as an RNA-binding protein involved in mRNA stability and translational modulation. It contains an Au-rich element present in 3′-UTR and neuronal differentiation [94]. The leading target mRNAs of HuD are growth-associated protein 43 (GAP43), acetylcholine transferase (AchE), p21, c-myc, N-myc, Notch3, VEGF-A, and so on [111]. Previously, HuD expression has been reported in small cell lung carcinoma and neuroblastoma [112,113]. In addition, we previously reported that HuD regulated the invasion ability and activation of caspase-3, and the main target genes in OSCC cells of HuD are *VEGF-A*, *VEGF-D*, *MMP-2*, and *MMP-9* [114]. In OSCC specimens, HuD expression was detected in 36.6% (30/82), and its expression closely correlated with the histological differentiation of the tumor, nodal metastasis, and diffuse invasion pattern. Moreover, a survival curve analysis revealed markedly worse outcomes in patients with the HuD expression than patients who were HuD-negative, and the HuD expression was an independent prognostic predictor in patients with OSCC. Besides, HuD is a useful diagnostic and therapeutic target in OSCC. As MMP-2 and -9 are components of the epithelial basement membrane and extracellular matrix proteins, HuD could be a novel modulator for the tumor microenvironmental modification in OSCC [114]. Furthermore, our results suggested that HuD is newly detected target of VEGF-A–mediated angiogenesis in OSCC.

### 3.6. STOX2

Storkhead box protein 2 (STOX2) is considered a transcriptional factor, and its expression is decreased in the decidual tissue of patients with fetal growth restriction [115]. A prior cDNA microarray analysis revealed that *STOX2* expression is related to prognosis in CRC [116]. Conversely, a study reported that *STOX2* expression levels in CRC were decreased by CpG island hypermethylation of the *STOX2* promoter region [95]. Hence, the role of STOX2 in malignancies remains unclear. In OSCC cells, STOX2 expression levels were increased by *MIA*, secretory protein of melanoma, in a paracrine manner [117]. In addition, STOX2 modulated the cell growth, invasion, and inhibition of apoptosis in OSCC cells by interacting with *MIA*. Moreover, STOX2 promoted resistance to paclitaxel, cisplatin, and 5-FU in OSCC. In fact, immunostaining of STOX2 was observed in 28.7% (58/202) of OSCC cases and associated with nodal metastasis, *MIA* expression, and poor survival. Multivariate analysis revealed that STOX2 expression was an independent predictor of disease-free survival in OSCC patients. Interestingly, STOX2 expression was also observed in the stromal plasma cells surrounding OSCC. Although further studies are warranted to validate STOX2’s role in the tumor stroma, it might contribute to the disruption of the host immune system. Hence, the *MIA*–STOX2 pathway might be a useful molecular target in OSCC.

### 3.7. N4BP2L1

Previously, we compared the gene expression profiles of primary and recurrent OSCC using cDNA microarray analysis, and the most increased level of expression in recurrent OSCC was NEDD4-binding protein 2-like 1 (*N4BP2L1*) [118]. Although N4BP2L1 is a critical paralog of N4BP2, highly expressed in nasopharyngeal carcinoma [119], little information was available about the functional role of N4BP2L1 in tumor cells. We determined that N4BP2L1 enhances invasion ability and miR-448 inversely regulates N4BP2L1 expression in OSCC cells [118]. In addition, the N4BP2L1 expression was observed in 34.8% (65/187) of OSCC cases by immunohistochemistry, and a marked correlation was observed between the N4BP2L1 expression and nodal metastasis. A gene expression analysis of 45 OSCC samples indicated lower *miR-448* expression levels were conversely associated with *N4BP2L1* upregulation. Moreover, the N4BP2L1 overexpression correlated with poor outcome and was an independent predictor of disease-free survival in OSCC patients. Hence, N4BP2L1 could be a new target for diagnosis and treatment of OSCC.

### 3.8. ZFAND4

Zinc finger AN1-type containing 4 (*ZFAND4*) is one of the most upregulated genes in recurrent OSCC samples [118]. Although higher *ZFAND4* expression, regulated by miR-182, strongly correlated with clinical stage progression in gastric cancer [120], little information is available about the functional roles of ZFAND4 in malignancies. Accordingly, we assessed the immunostaining of ZFAND4 in 214 OSCC cases [121]. The cytoplasmic expression of ZFAND4 was detected in 21% (45/214) of OSCC cases, and there appears to be a link between ZFAND4 expression and lymph node metastasis, lymphatic invasion, vascular infiltration, and poorer outcome. In addition, the ZFAND4 overexpression was considered an independent predictor of unfavorable prognosis in OSCC cases as revealed by a multivariate analysis. Intriguingly, high ZFAND4 expression was also implicated in distant OSCC metastasis. While 3.8–12.6% OSCC patients experience metastasis, the disease becomes highly lethal when metastasis occurs [121,122]. Hence, ZFAND4 could be an essential molecular marker and therapeutic target for distant metastasis and prognosis of OSCC cells.

### 3.9. NIPAL1

Previously, we identified NIPA-like domain containing 1 (*NIPAL1*) as an overexpressed gene in recurrent OSCC [118]. *NIPAL1* is a membranous magnesium transporter and has been associated with the pathogenesis of gout and hyperuricemia by indirect urate transport regulation [123]. Research has demonstrated that hyperuricemia is associated with an increased risk of cancer [124]. However, little is known about the *NIPAL1*’s role in malignancies. In OSCC cells, *NIPAL1* accelerated cancer cell proliferation and adhesion to vascular endothelial cells (intravasation) [125]. However, *NIPAL1* failed to affect the transendothelial migration, tube formation, and branching of endothelial cells. Perhaps *NIPAL1* might merely accelerate OSCC cell infiltration that had been evoked by other angiogenic factors. The *NIPAL1* expression was detected in 20.3% (39/192) of OSCC cases and correlated strongly with vascular invasion and short disease-free survival. Furthermore, the *NIPAL1* expression was an independent predictor of poor prognosis in OSCC patients.

### 3.10. LEMD1

LEM domain containing 1 (*LEMD1*) comprises several splicing variants, and LEMD1 variant 1 (V1), V2, and V3 are cancer-testis antigens (CTA) [126]. Reportedly, LEMD1 overexpression has been detected in colon cancer, prostate cancer, and anaplastic large-cell lymphoma [126,127,128]. In addition, immunostaining for LEMD1 has been reported in 35% (101/289) of OSCC specimens and closely involved in local progression (T factor), clinical stage, and nodal metastasis [129]. The disease-free survival among all LEMD1-positive patients was considerably worse compared to LEMD1-negative patients, and the LEMD1 expression was an independent prognosticator. In an in vitro analysis using OSCC cells, LEMD1 enhanced invasion ability. Moreover, we determined that LEMD1 controlled the intravasation and transmigration of OSCC cells to endothelial cells. Since CTA in cancer is a useful target of immunotherapy through the activation of CTL [130], LEMD1 normalization might be useful for activating the host immune function of OSCC. Perhaps, LEMD1 might be a novel tumor-promoting and prognostic CTA that induces the gain of invasion ability and transendothelial migration of OSCC.

### 3.11. PAUF

Pancreatic adenocarcinoma upregulated factor (PAUF) is a newly determined secretory protein in pancreatic cancer [131]. PAUF is a ligand for toll-like receptor 2 (TLR2) and TLR4 and can promote the migration, invasion, proliferation, angiogenesis, and CXC receptor type 4 (CXCR4)–mediated metastasis of pancreatic cancer cells [131,132,133,134]. In addition, PAUF contributes to the insufficiency of T-cell immunosurveillance and immunoescape through the migration and activation of myeloid-derived immature cells in pancreatic cancer [135]. Moreover, PAUF has been reported to decrease pancreatic cancer cells’ sensitivity to gemcitabine and 5-FU [134]. Recently, we reported that PAUF facilitated growth, invasion, suppression of apoptosis, and cisplatin resistance in OSCC cells [136]. In an immunohistochemical analysis, PAUF expression was detected in 23.4% (52/222) of OSCC cases, and the immunoreactivity for PAUF markedly correlated with nodal metastasis. We also revealed that PAUF-positive patients exhibited a remarkably shorter disease-free and overall survival than PAUF-negative patients. Furthermore, a multivariate analysis revealed that PAUF expression was an independent prognostic predictor of poor disease-free survival and cancer-specific mortality of OSCC patients. Thus, our findings indicate that PAUF is a useful molecular target for OSCC diagnosis and therapy.

### 3.12. ME1

Malic enzyme 1 (ME1) is a multifunctional protein involved in glycolysis, the citric acid cycle, NADPH production, glutamine metabolism, and lipogenesis [137]. In malignancies, ME1 overexpression correlated with unfavorable prognosis in HCC patients by EMT induction [137]. In addition, ME1 is associated with tumor growth, lung metastasis, peritoneal dissemination, and shorter overall and disease-free survival in gastric cancer cases [138]. Our experimental data suggested that ME1 promotes cancer progression by increasing lactate fermentation, maintaining redox status, acquiring stemness and EMT phenotype, and promoting tumor growth and invasion in OSCC cells [74]. In addition, ME1 expression closely correlated with local progression (T factor), clinical stage, and nodal metastasis in human OSCC specimens. Furthermore, the survival analysis using the Kaplan–Meier method revealed that cases with moderate-to-strong ME1 expression exhibited markedly worse prognosis than those with weak ME1 expression. Since inhibiting ME1 suppressed tumor growth and increased survival time in a mouse tumor model, ME1 could be a valid target for molecular therapy in OSCC.

## 4. Conclusions

Initially, Hanahan and Weinberg proposed six hallmarks of the cancer cell model: sustained proliferative signals, evasion of growth suppressors, resistance to cell death, replicative immortality, induction of angiogenesis, and activation of invasion and metastasis [6,139]. Considering the dramatic progress in modern cancer research, they added two emerging hallmarks—avoidance of immune destruction and deregulation of cellular energetics—and two enabling characteristics—genome instability and mutation and tumor-promoting inflammation—in 2011 [5,6]. Although advances in molecular oncological biology have elucidated OSCC molecular mechanisms, the prognosis of locoregionally and metastatically advanced cancer awaits improvement. Several studies about invasion, metastasis, and prognosis-related molecular biomarkers for malignancies, including OSCC, have been published to date. Recently, molecular-targeted therapy using cetuximab, an anti-EGFR-specific chimeric monoclonal antibody, and nivolumab, an antibody inhibitor of PD-1 receptor, is used in OSCC patients [140,141]. However, other targets for diagnosis and treatment of OSCC remain unknown, necessitating the development of useful molecular tumor markers. Hopefully, relevant novel tumor biomarkers will be established in the near future.

## Figures and Tables

**Table 1:** Functional roles of novel prognosticators of oral squamous cell carcinoma (OSCC).

Factors	Behavior	Role on the Hallmarks of Cancer	References
TANGO	Upregulated	Sustaining proliferative signaling Activating invasion and metastasis Inducing angiogenesis Resisting cell death	[46]
ME1	Upregulated	Sustaining proliferative signaling Activating invasion and metastasis Deregulating energetics	[58]
miR-126	Downregulated	Sustaining proliferative signaling	[68]
		Evading growth suppressors	
		Inducing angiogenesis	
FOXC2	Upregulated	Inducing angiogenesis	[75]
PROX1	Upregulated	Sustaining proliferative signaling	[75]
		Activating invasion and metastasis	
		Inducing angiogenesis	
HuD	Upregulated	Activating invasion and metastasis	[89]
		Resisting cell death	
STOX2	Upregulated	Sustaining proliferative signaling	[90]
		Avoiding immune destruction	
		Activating invasion and metastasis	
		Resisting cell death	
N4BP2L1	Upregulated	Activating invasion and metastasis	[91]
ZFAND4	Upregulated	Activating invasion and metastasis	[92]
		Inducing angiogenesis	
NIPAL1	Upregulated	Inducing angiogenesis	[93]
LEMD1	Upregulated	Activating invasion and metastasis	[94]
		Inducing angiogenesis	
PAUF	Upregulated	Sustaining proliferative signaling	[95]
		Activating invasion and metastasis	
		Inducing angiogenesis	
		Resisting cell death	
ME1	upregulated	sustaining proliferative signaling	[58]
		activating invasion and metastasis	
		deregulating energetics	

TANGO: transport and Golgi organization protein 1; ME1: malic enzyme 1; FOXC2: forkhead box protein C2; PROX1: prospero homeobox 1; HuD: Hu antigen D; STOX2: Storkhead box protein 2; N4BP2L1: NEDD4-binding protein 2-like 1; ZFAND4: zinc finger AN1-type containing 4; NIPL1: NIPA-like domain containing 1; LEMD1: LEM domain containing 1; PAUF: pancreatic adenocarcinoma upregulated factor.

## Abbreviations

5-Aza-dc	5-aza-2′-deoxycytidine
5-FU	5-fluorouracil
AchE	acetylcholine transferase
ATP	adenosine triphosphate
Bak	Bcl-2 homologous antagonist killer
Bax	Bcl-2-associated X protein
Bcl-2	B-cell lymphoma-2
COX-2	cyclooxygenase-2
CRC	colorectal cancer
CTL	cytotoxic T cells
CXCL9	chemokine (C-X-C motif) ligand 9
CXCR4	CXC receptor type 4
*DCRG8*	Drosha and DiGeorge syndrome critical region gene 8
EGF	epidermal growth factor
EGFL7	epidermal growth factor-like domain 7
EGFR	epidermal growth factor receptor
EMT	epithelial–mesenchymal transition
FasL	Fas ligand
FDG-PET	[^18^F]fluoro-2-deoxyglucose-positron emission tomography
FOXC2	Forkhead box protein C2
GAP43	growth-associated protein 43
GLUT-1	glucose transporter 1
HER1	human epidermal growth factor receptor 1
HIF1-α	hypoxia-inducible factor 1a
hTERT	human telomerase reverse transcriptase
HuD	Hu antigen D
IL	interleukin
IRS-1	insulin receptor substrate 1
LEMD1	LEM domain containing 1
LOH	loss of heterozygosity
MAPK	mitogen-activated protein kinase
ME1	malic enzyme 1
*MIA2*	melanoma inhibitory activity 2
miRNAs	microRNAs
MMP	matrix metalloproteinase matrix
mTOR	mammalian target of rapamycin
*N4BP2L1*	NEDD4-binding protein 2-like 1
*NIPAL1*	NIPA-like domain containing 1
NK	natural killer
OSCC	oral squamous cell carcinoma
PAUF	pancreatic adenocarcinoma upregulated factor
PD-1	programmed cell death 1
PDGF	platelet-derived growth factor
PDGFB	platelet-derived growth factor-β polypeptide
pEGFR	phosphorylated EGFR
PI3K	phosphoinositide 3-kinase
POT1	protection of telomeres
pre-miRNA	precursor miRNA
pri-miRNA	primary miRNA
PROX1	prospero homeobox 1
pSTAT3	phosphorylated STAT3
PTEN	phosphatase and tensin homolog
RAP1	repressor activator protein 1
RISC	RNA-induced silencing complex
SH3	Src homology 3
SIS	systemic inflammatory score
Spred-1	sprouty-related protein-1
STAT	signal transducer and activator of transcription
STOX2	Storkhead box protein 2
*TANGO*	Transport and Golgi organization protein 1
TGF-β	transforming growth factor-β
Th1 cells	T helper type 1 cells
TIN2	TRF1-interacting nuclear factor 2
TLR2	toll-like receptor 2
TNFα	tumor necrosis factor-α
TPP1	POT1-interacting protein
TRAIL	TNF-related apoptosis-inducing ligand
Treg	T regulatory
TRF	telomeric repeat factor
UTR	untranslated region
VEGF	vascular endothelial growth factor
*ZFAND4*	zinc finger AN1-type containing 4
